# Special Issue “Laser Powder Bed Fusion, Direct Energy Deposition and Hybrid Manufacturing of Metals and Alloys”

**DOI:** 10.3390/ma15196990

**Published:** 2022-10-08

**Authors:** Hector R. Siller

**Affiliations:** Department of Mechanical Engineering, University of North Texas, 3940 N. Elm. Str., Denton, TX 76207, USA; hector.siller@unt.edu

Hybrid additive manufacturing processes involve the use of different manufacturing techniques to fabricate net shape or near-net shape parts, with enhanced capabilities of heat dissipation, such as those needed in conformal molding, or requiring internal cooling systems, such as, for example, those seen in turbine blades, and for developing other components demanding free form fabrication methods. The different techniques include additive manufacturing (AM), specifically the different processes defined in the classification presented in the ISO/ASTM 52900 standard for metallic materials for single-step and multi-step AM processing principles [1]. Among the most well-known are powder-bed fusion, direct energy deposition, binder jetting and sheet lamination. The combination of these AM processes with material removal processes, such as machining, is the core of the hybrid additive manufacturing concept, together with the full coupling of these processes in an integrated fashion [2]. 

Laser powder bed fusion (L-PBF) and direct energy deposition (DED) are versatile additive manufacturing processes with the capability to produce high-quality parts at high productivity rates and can be combined with subtractive manufacturing to improve surface characteristics. The industry is exponentially adopting these additive and hybrid manufacturing processes to fabricate functional parts for structural, biomedical, aerospace, and automotive applications, among others. Nevertheless, the introduction of additively manufactured parts is still experiencing barriers to reach acceptable levels of product integrity, with high-performance functions and under strict service requirements. For example, the manufacturing of certain parts of aeronautical structures is still far from providing good reliability, and said parts are prone to failure under certain fatigue and stress conditions. The causes are several, but among them, one can count the lack of fusion at different regions of the build, poor surface finish and excess of porosity, even after post-treatment processes such as hot isostatic pressing (HIP). The aim of this Special Issue is to collect valuable research in different fields affecting the process and product integrity in additive and hybrid manufacturing processes of metals and alloys, focusing on surface integrity, crystallography evolution, porosity, anisotropy effects, process calibration and laser–material interaction effects. Other research fields are welcome if their approach is related to hybrid manufacturing processes and product enhancement.

There are other different opportunities to contribute to this Special Issue, in the form of original manuscripts involving different aspects of additive and hybrid manufacturing processes, not only involving actual process chains, but also incorporating feedstock and powder characterization, development of novel post-processing techniques and the fabrication of test-beds for materials qualification among other research topics. Industrial case studies are welcome to provide process planning guidelines to academic researchers and practitioners, with the ultimate goal of assessing process viability in the long term, given the considerable amount of energy put into the field of free form manufacturing techniques in recent years. 
